# Bridging the gap of vision restoration

**DOI:** 10.3389/fncel.2024.1502473

**Published:** 2024-11-21

**Authors:** Maya Carleton, Nicholas W. Oesch

**Affiliations:** ^1^Department of Psychology, University of California San Diego, La Jolla, CA, United States; ^2^Department of Ophthalmology, University of California San Diego, La Jolla, CA, United States; ^3^Neuroscience Graduate Program, University of California San Diego, La Jolla, CA, United States

**Keywords:** vision restoration, retinal degeneration, age related macular degeneration, retinitis pigmentosa, retinal prosthesis, retina, optogenetics

## Abstract

Retinitis pigmentosa (RP) and Age-Related Macular Degeneration (AMD) are similar in that both result in photoreceptor degeneration leading to permanent progressive vision loss. This affords the possibility of implementing vision restoration techniques, where light signaling is restored to spared retinal circuitry to recreate vision. There are far more AMD patients (Wong et al., 2014), yet more resources have been put towards researching and developing vision restoration strategies for RP despite it rarity, because of the tractability of RP disease models. The hope is that these therapies will extend to the AMD population, however, many questions remain about how the implementation of prosthetic or optogenetic vision restoration technologies will translate between RP and AMD patients. In this review, we discuss the difference and similarities of RP and AMD with a focus on aspects expected to impact vision restoration strategies, and we identify key gaps in knowledge needed to further improve vision restoration technologies for a broad patient population.

## 1 Introduction

**Figure 1:**
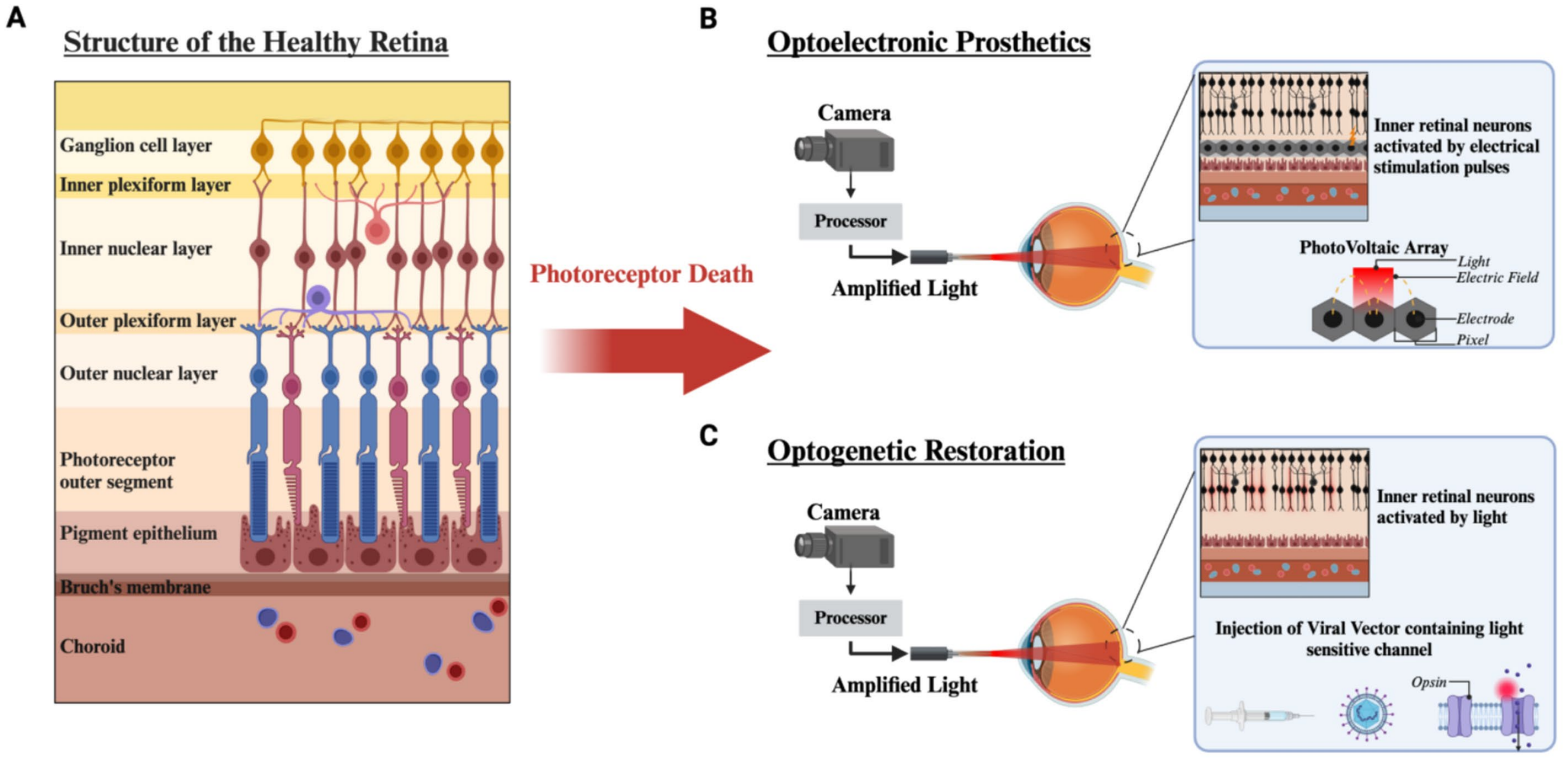
Structure of the retina and vision restoration strategies after photoreceptor degeneration. **(A)** The structure of the healthy retina is laminated into distinct layers. After photoreceptor loss due to RP or AMD, vision restoration strategies can be implemented. **(B)** Optoelectronic prosthetics utilize a camera mounted to external hardware and a processor which amplifies natural light and projects Near Infrared light (NIR) into the eye onto a photovoltaic array to generate stimulating current through a microelectrode array. **(C)** Optogenetic restoration uses a viral vector to introduce a light sensitive ion channel targeted to bipolar or ganglion cells. Utilizing a similar external setup to photovoltaic optoelectronics, light from the environment is amplified to obtain sufficient activation of the light sensitive molecule. Created with www.biorender.com.

The retina is a part of the central nervous system responsible for the first steps in vision. It consists of light sensitive cells, called photoreceptors, interneurons for processing visual information and retinal ganglion cells whose axons make up the optic nerve and send visual signals to the brain (Figure 1A). Retinal Degenerative Diseases (RDD) are the leading cause of blindness in humans, with Age-Related Macular Degeneration (AMD) being the most common. AMD currently affects 196 million people throughout the world, with the prevalence expected to increase as the global population ages (Wong et al., 2014; Bourne et al., 2017). In contrast, Retinitis Pigmentosa (RP) is a RDD with a smaller clinical population of around 2 million globally, however, it is still the most prevalent cause of inherited blindness (Garip and Kamal, 2019). Both diseases ultimately result in the loss of photoreceptors leading to local or global blindness, and no cure exists for the vast majority of cases.

One strategy to restore vision in these patients is to replace the light sensing photoreceptor vision with an artificial phototransducing technology. Currently, there are two main strategies, optoelectronic visual prostheses, and optogenetics. Optoelectronic prosthesis utilizes implantable semiconductor based light sensors coupled to stimulating microelectrodes to excite neurons downstream of the photoreceptors. These can be placed between the pigment epithelium and remaining bipolar cells (subretinal) (Muqit et al., 2024), implanted into the suprachoroidal space (Karapanos et al., 2021) or placed above retinal ganglion cells (epiretinal) (Luo and da Cruz, 2016) (Figure 1B). In contrast, the optogenetic approach uses gene therapy vectors to introduce non-native light sensitive proteins into spared cells of the retina bipolar cells (Wood et al., 2023), or ganglion cells (McGregor et al., 2020) (Figure 1C). Both technologies are being used in clinical trials, but no one therapy is targeting both diseases, and currently no vision restoration technology is available in the marketplace.

Vision restoration strategies have primarily targeted RP despite the smaller patient population, because late-stage RP patients with bare or no light perception have little risk for worsening vision. This strategy limits the accessibility of vision restoration therapies to the relatively small population of patients with late-stage RP (Walsh et al., 2018). The common hope is that if a vision restoration technology can show safety and efficacy in an RP patient population, it may be translated to AMD patients in the future, unlocking a much larger patient population. However, the pre-clinical work, remains incomplete.

Although reintroducing light sensitivity to the retina appears generally feasible, a major challenge is that spared retina can undergo structural and functional plasticity following the degeneration of photoreceptors, which can affect downstream signaling. How inner retinal changes in RP and AMD are similar or different is still unknown Therefore, understanding the degree to which these changes impact the restoration of visual signals will crucially influence the degree of success in vision restoration. Here we provide a review of RP and AMD, to understand the functional and anatomical changes in both diseases. Given that most preclinical vision restoration work is done in the context of RP, while the larger patient population lies in AMD, the knowledge of where the RP literature can be leveraged to aid in AMD, and where the diseases differ will be paramount to bringing vision restoration strategies to the broader RDD patient population. Here, we highlight current gaps in knowledge that are important for the implementation of high-fidelity vision restoration in patients with both RP and AMD.

## 2 Retinitis pigmentosa

### 2.1 Etiology

Retinitis Pigmentosa is comprised of a class of heterogeneous gene mutations and phenotypes (Van Soest et al., 1999). Mutations can be classified as non-syndromic, affecting the retina in isolation, or syndromic in which retinal degeneration coincides with other disease processes that occur outside the eye (Hamel, 2006). The most notable syndromic diseases are Usher syndrome and Bardet-Biedl syndrome, but there are approximately thirty syndromes coincident with RP (Phelan and Bok, 2000).

Non-syndromic retinitis pigmentosa is a class of inherited retinal dystrophies that present without other systematic abnormalities (Pierrottet et al., 2014). Inherited non-syndromic RPs are composed of autosomal dominant (15–20%), autosomal recessive (5–20%), X-linked (5–15%), and a vast majority of unknown genetic patterns (40–50%) (Fahim et al., 1993). Eighty-four gene mutations have been identified so far, affecting a wide range of biological functions, which ultimately result in the death of rod photoreceptors. Principal genes affect the visual transduction cascade, visual cycle, and ciliary function and transport within rod photoreceptors (Verbakel et al., 2018).

**Figure 2:**
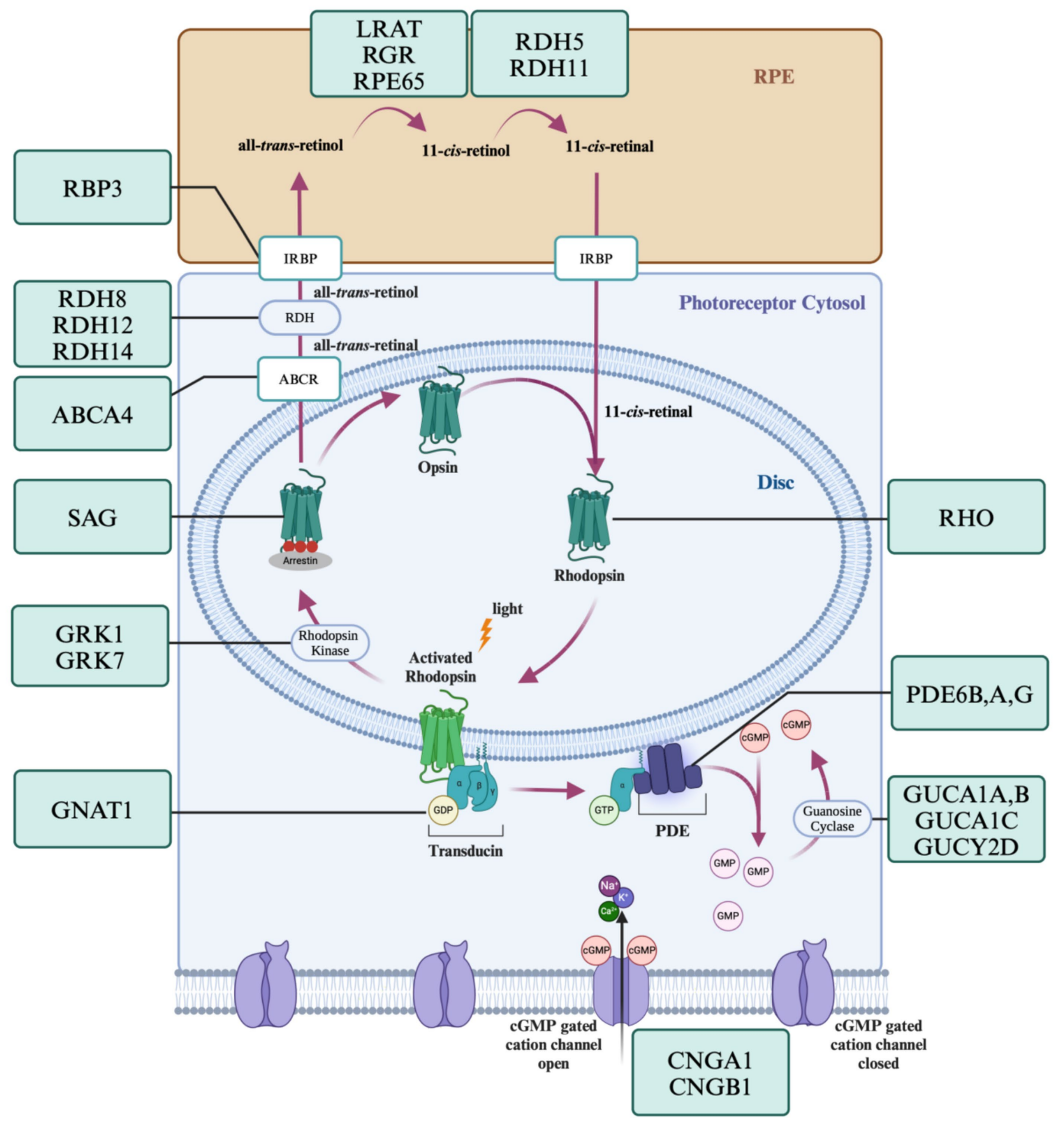
Genes of the visual transduction and visual cycle known to cause RP. The phototransduction cascade and visual cycle are a tightly regulated series of steps requiring the correct function of many proteins. Mutations in the genes encoding these proteins can lead to apoptosis via endoplasmic reticulum stress, excitotoxic levels of cGMP, excitotoxic levels of intracellular calcium, and continuous phototransduction. Created with www.biorender.com.

Figure 2 displays some genes in the visual transduction cascade and photopigment cycle that when disrupted result in RP. Almost every step in the phototransduction cascade and cycle when disrupted could potentially result in rod death and the development of RP. There are four dominant pathways to apoptosis in rods; mutations that create endoplasmic reticulum (ER) stress, excitotoxic levels of cGMP, excitotoxic levels of intracellular calcium, and continuous phototransduction (Koenekoop, 2011; Newton and Megaw, 2020). *RHO* mutations are the most common mutations in RP and many affect protein folding. Excess accumulation of misfolded proteins in the ER can result in stress and ultimately the activation of apoptotic pathways via caspases (Comitato et al., 2020). Gene mutations such as those to the phosphodiesterase subunits results in excitotoxic levels of cGMP as they are no longer hydrolyzed to 5’ GMP and increases intracellular calcium (Barabas et al., 2010). Finally, mutations in genes such as *RPE65* and *GNAT1* result in the continuous activation of the phototransduction cascade (Hao et al., 2002), recruiting similar apoptotic pathways to those observed in light damage.

Ciliopathies, which affect ciliogenesis and ciliary protein trafficking, can have detrimental effects on many bodily organs including photoreceptors which are specialized sensory cilia. Rod-cone atrophy in both Usher Syndrome and Bardet-Biedl Syndrome are thought to be caused by deficits in the trafficking between the rod inner and outer segments due to mutated cilia (Waters and Beales, 2011; Tsang et al., 2018; Forsythe and Beales, 2013). Genes encoding trafficking proteins such as *MYO7A* (Williams and Lopes, 2011), *PCDH15* (Ahmed et al., 2003), and *USH2A* can have profound adverse effects on photoreceptors when disrupted, such as, impaired intracellular directional transport, impaired outer segment formation (Özgül et al., 2011), mislocalized proteins (Zhang et al., 2015), and misrouting of opsins (Jacobson et al., 2008; Reiter and Leroux, 2017). The pathways to apoptosis of ciliopathies are not fully understood but may also be due to ER stress. In many cases multiple pathways to apoptosis may be recruited or are not completely known.

Cone death is secondary to that of rods and follows rod death. The factors leading to cone degeneration have remained somewhat elusive, but there are four prevailing hypotheses. First, rods typically provide the trophic survival factor, rod-derived cone viability factor (RdCVF), to cones to promote survival (Léveillard et al., 2004). The reduction in RdCVF as rods degenerate could reduce the viability of cones via diminished glucose uptake. The second hypothesis is the ‘Cone Starvation’ hypothesis. Proposed by Punzo et al. (2009), they found the mTOR pathway which is typically involved in mediating anabolic processes of cells is diminished due to low nutrient conditions, and promotes the autophagy of cones. They identified upregulation of GLUT-1 in cones, as a compensatory mechanism for the decrease in glucose. Thus, the first two hypothesis converge on a pathway involving glucose loss and cone starvation. The third hypothesis involves the increase in oxidative stressors called reactive oxygen species (ROS). Cones are especially susceptible to oxidative stress due to their high metabolic load (Shen et al., 2005; Hoang et al., 2002). Within the retina, rods consume 95% of the oxygen delivered by the choroid and as rods degenerate the choroid has no mechanism to autoregulate oxygen levels (Yu et al., 2000). Increasing oxygen results in ROS species, which cause mitochondrial breakdown ultimately leading to apoptosis (Kevany and Palczewski, 2010). Finally, microglia activation coincident with rod death may influence cone apoptosis (Gupta et al., 2003; Zeiss and Johnson, 2004). Microglia recruitment is a normal function of the inflammatory immune response to damage, but in many cases, it has both protective and destructive effects (Block and Hong, 2005; Graeber and Streit, 2010). Under these circumstances microglia release pro-inflammatory cytokines that can bind to cones and activate proapoptotic pathways (Zeng et al., 2005). Evidence suggests that these pathways work in concert to produce cone degeneration.

### 2.2 Clinical presentation

Many identified genes causing RP affect the visual transduction cascade in rods, where mutations in many steps in phototransduction can result in photoreceptor death (Phelan and Bok, 2000). As rods are the first to degenerate, patients first experience night blindness, before peripheral vision loss, and the transition from partial to complete blindness results from secondary loss of cones following rod degeneration. The clinical presentation of RP typically takes decades to evolve, and for much of the early stages, patients may not notice progressive visual changes. The diagnostic criterion for RP encompasses night blindness, peripheral field deficits, lesions within the fundus, and hypovolted electroretinogram (ERG) (Michael and Fletcher, 2004; Hamel, 2006; Kaplan et al., 1990; Baumgartner, 2000; Li et al., 1995). In the early stages of RP, changes in the ERG may be the first indicator that a patient is developing RP, as the fundus can appear clear of pigmentary deposits and the patient may not report any perceptual changes to their vision.

As the disease progresses to intermediate stages, patients begin to notice worsening night vision, culminating in the inability to drive at night or navigate in dim light. In addition, patients also experience visual changes under photopic light conditions. Scotomas can appear, creating blind spots in the peripheral visual field (Grover et al., 1998). Patients may also become photophobic, especially under diffuse lighting conditions, such as bright cloudy weather (Otsuka et al., 2020). Color vision can be disrupted leading to dyschromatopsia specifically in blue/yellow color perception (Jeffrey et al., 2014). Often patients do not seek help until their central visual acuity is compromised due to extensive cone loss, and they are no longer able to read. In this stage, the scotopic ERG is nonexistent and the photopic ERG is significantly reduced (Nagy et al., 2008). Fundoscopy images show bone-spicule shaped deposits in the periphery and narrowing of retinal vasculature (Li et al., 1995).

In the late stages of RP, patients are left with very little, if any, foveal vision. The fundus shows dramatic bone spicule pigmentation, thin vasculature, and optic disk discoloration. The choroid also begins to atrophy in the periphery and macular regions. With little to no vision remaining, this is the stage in which retinal prosthesis have traditionally been implemented to restore vision, given the low risk to further vision loss. Prior work shows that patients with late stage RP have a harder time using assistive technologies limiting their ability to pursue higher education and maintain jobs (Chaumet-Riffaud et al., 2017). As such, vision restoration could have a large impact on patients’ quality of life as they regain the ability to independently navigate. However, extensive disease mediated plasticity in the inner retina may also limit the potential quality of restored vision. These considerations demonstrate the importance for gauging the best disease stage to implement vision restoration strategies for both RP and AMD from a cost/benefit perspective.

Given the relatively simple genetic origins of most RP, gene therapies have garnered much excitement for the potential to “cure” RP by delivering the required gene to correct rod dysfunction before degeneration begins. Nevertheless, only one gene therapy has been approved by the FDA (Luxturna, Spark Therapeutics), which is approved for use in patients with Leber Congenital Amaurosis. Luxturna targets the RPE65 gene and showed improvements in the majority of patients at follow-up. While Luxturna paved the way for gene therapies it only targets 0.3–1% of RP cases (Wu et al., 2023), and other gene therapies remain in the clinical trial stage. As others have reviewed the current state of gene therapies for inherited retinal diseases, we direct interested readers to Amato et al. (2021), for a more in depth discussion.

Other therapies are under development to slow the progression of RP such as injections of Rod Cone viability factor (Yang et al., 2009), glucose addition (Kaplan et al., 2021), or the anti-inflammatory drug dexamethasone (Guadagni et al., 2019). Interestingly, these trophic drugs may also be useful for AMD as rod loss precedes cone loss and metabolic stress is a feature of both diseases. However, any benefit is only afforded after the loss of rods, and may only slow, not prevent, the loss of cones.

## 3 Age related macular degeneration

### 3.1 Etiology

The etiology and pathophysiology of AMD are more complex and less well understood than RP, but several factors significantly increase the incidence of developing AMD. While no one gene or factor will in isolation result in AMD, the largest factor contributing to disease is age. Other environmental factors include smoking (Klein et al., 1993a,b; Smith et al., 1996), diet (Chapman et al., 2019), hypertension (Hogg et al., 2008; Katsi et al., 2015), oxidative stress (Beatty et al., 2000; Shaw et al., 2016; Jarrett and Boulton, 2012), alcohol intake (Chapman et al., 2019), BMI, and sunlight exposure (Age-Related Eye Disease Study Research Group, 2000; Coleman et al., 2008), while traits not directly associated with disease progression include race (Friedman et al., 1999; Klein et al., 1999), iris color (Cruickshanks et al., 1993; Taylor et al., 1992), and gender (Mousavi and Armstrong, 2013). The degree to which genes result in AMD and the severity of progression is not completely understood. However, a risk factor scale has been developed using additive odds ratios for each individual risk factor. Risk categories are low (1.0–7.90), average (8.0–28.9), moderate (29.0–100), high (101–184), severe (185–2,600) (Zanke et al., 2010). With increasing risk score, patients increase the prevalence of progressing to the late-stage AMD outcomes, geographic atrophy and choroidal neovascularization. Primary genes include *CFH*, *ARMS2*, *C3*, *CFB*, and *C2*, with risk scores of 7.2, 25.4, 3.6, 0.3, and 0.3, respectively, (Seddon et al., 2009).

#### 3.1.1 Dry AMD

**Figure 3:**
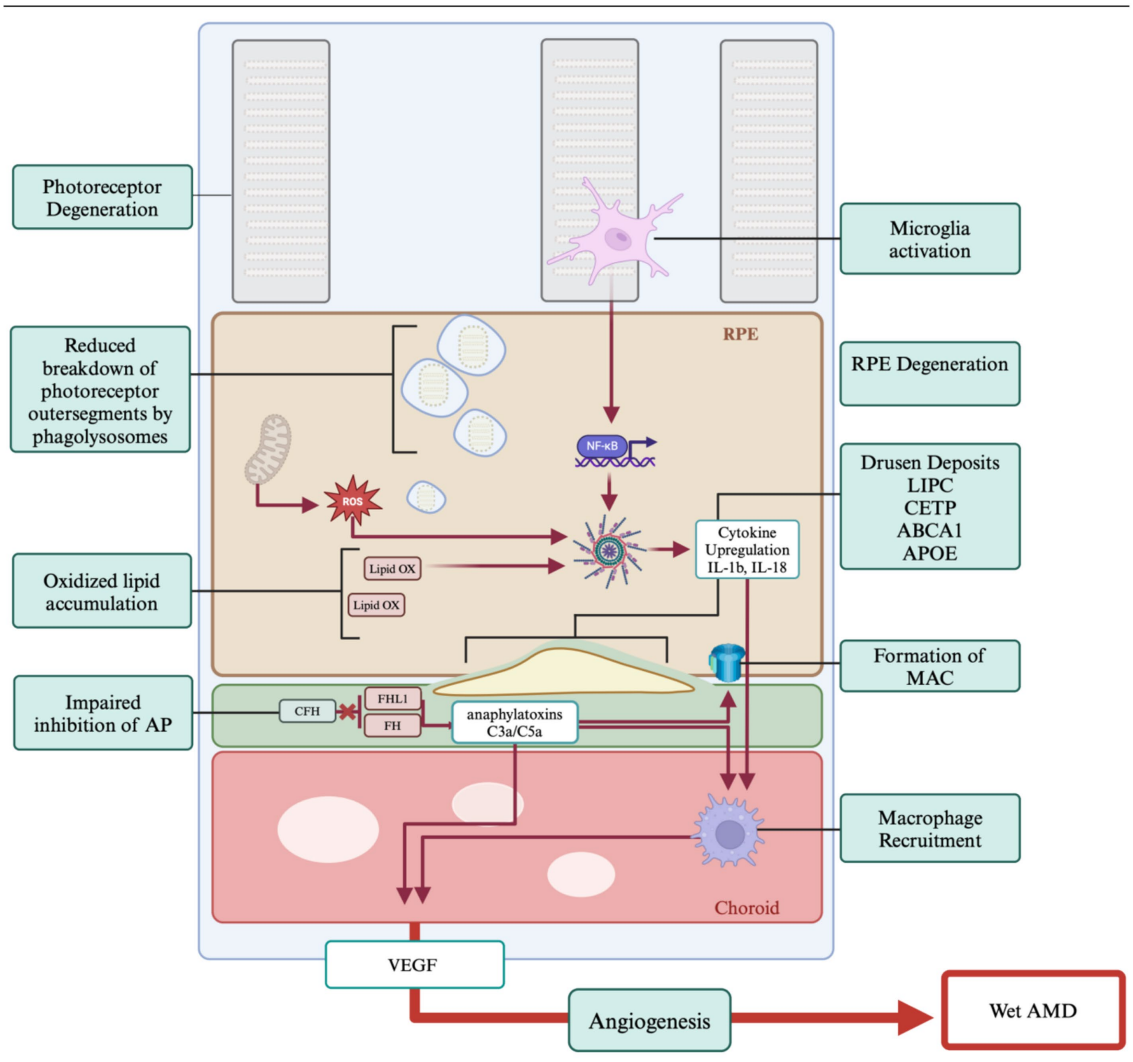
Genes and pathogenesis of AMD. The primary hallmark of AMD is the formation of drusen which results due to impaired cholesterol transport. Age and oxidative stressors play a significant role in AMD progression. Chronic inflammation is tied to the immune response which is impaired in AMD. Proangiogenic pathways lead to the development of wet AMD. Created with www.biorender.com.

The retina is one of the most metabolically expensive organs in the body (Yu and Cringle, 2001; Joyal et al., 2018). The function of over 91 million rods and 4.5 million cones are dependent upon proper nutrient access and waste product disposal through the RPE (Young and Bok, 1969; Lakkaraju et al., 2020). RPE cells are non-renewing and as we age, waste products begin to accumulate. Photoreceptors shed approximately 10% of their outer segment volume a day, which must be broken down by the RPE and the waste products trafficked out through Bruch’s Membrane and into the choroid (Kevany and Palczewski, 2010; Young, 1971). Certain byproducts of this process are more difficult to breakdown, such as lipofuscin, which is produced by incomplete digestion of outer segments by phagolysosomes (Wang et al., 2009; Keeling et al., 2018). As lipofuscin accumulates in the RPE it is converted into N-retinylidene-N-retinylethanolamine (A2E), RPE cells with high A2E generate high levels of reactive oxygen species (ROS), hydrogen peroxide and super oxide, which are toxic to RPE cells and promotes their degeneration (Moreno-García et al., 2018). Reactive oxygen species can further impair cellular respiration by causing mitochondrial breakdown, reducing both RPE and photoreceptor cell viability (Kohen and Nyska, 2002; King et al., 2004) (Figure 3).

Drusen deposits form between the RPE and Bruch’s Membrane and can be classified as hard or soft (Khan et al., 2016). While hard drusen are a normal part of ageing, soft drusen deposits can be the first indicator of AMD (Pugazhendhi et al., 2021). The causes of these deposits are multifaceted, with factors including inflammation, oxidative stress, and various genetic components (Coleman et al., 2008; Friedman et al., 2004), and the shift to larger drusen bodies accompanying AMD is not fully understood. These accumulations are primarily composed of lipids, specifically cholesterol, and there are several high-density lipoprotein associated genes implicated in AMD drusen formation identified through genome wide association studies (GWAS) in human patients (Rajanala et al., 2023). These genes are *LIPC*, *CETP*, *ABCA1*, and *APOE* which are involved in lipid and cholesterol transport and breakdown mechanisms (Storti et al., 2017). Disruption of the normal function of these genes leads to impairment of the cholesterol transport of the retina resulting in drusen formation and inflammatory signaling (Karlstetter et al., 2015; Campbell and Doyle, 2013).

The complement factor pathway has been strongly implicated in the pathobiology of AMD (Mousavi and Armstrong, 2013; Shaw et al., 2012; Lorés-Motta et al., 2018). Also known as the complement cascade, it acts through a series of protein interactions enhancing the clearance of damaged cells via the immune system. There are three pathways by which the complement cascade can be activated, the classical pathway (via antigen–antibody complexes), the alternative pathway (via spontaneous C3 hydrolysis), and the lectin pathway (via Mannose-binding lectin/MBL-associated serine proteases (MLB-MASP) complexes) (Merle et al., 2015). Dysfunction in the inhibition of the alternative pathway leads to over activation of the immune response and RPE cell death. GWAS studies have shown a significant role for the Complement factor H (*CFH*) gene in AMD (Shaw et al., 2012; Lorés-Motta et al., 2018). Since CFH inhibits the complement cascade, mutations in the gene may lead to increased anaphylatoxins, C3a and C5a, and C5 convertase which generates membrane attack complexes (MAC) thereby causing cell lysis (Parente et al., 2017).

#### 3.1.2 Choroidal neovascularized AMD

While choroidal neovascularized (CNV) AMD, commonly referred to as wet AMD, is less common than non-neovascularized AMD it is responsible for 90% of blindness in patients. In wet AMD, blood and/or serum leak into the subretinal space due to changes in the vascularization of the choroid. As wet AMD is a progression of dry AMD, many of the factors promoting vascularization originate from the dry pathobiology. Increases in inflammatory factors and dysfunction of the complement pathway eventually leads to the production of pro-angiogenic factors, however the reason not all AMD cases progress to CNV is not well understood. There are three primary angiogenic factors that are thought to cause vascularization that most likely work together, recruitment of macrophages, vascular growth factors, and vascular endothelial growth factors (Pugazhendhi et al., 2021).

First, macrophages are recruited due to immune response and can be pro-inflammatory (M1) and anti-inflammatory (M2) (Epelman et al., 2014). Research has shown that both types of macrophages are recruited in the development of CNV, however shifts in macrophage polarization from M1 to M2 resulted in increased angiogenesis (Jetten et al., 2014; Zandi et al., 2015). In studies where macrophages were depleted CNV was reduced but not eliminated (Espinosa-Heidmann et al., 2003). However, in mouse models that possess macrophage deficiencies CNV was shown to develop (Ambati et al., 2003). Thus, the role of macrophages in AMD is still unclear.

Second, the hormone angiopoietin 2 (Ang2) is found in elevated levels of patients with nvAMD (Ng et al., 2017). Ang2 is an antagonist for the transmembrane receptor Tyrosine kinase with immunoglobulin-like and epidermal growth factor-like domains 2 (Tie2). High levels of Ang2 antagonize Tie2 and have been shown to result in abnormal vasculature growth and permeability (Maisonpierre et al., 1997). Finally, vascular endothelial growth factors (VEGF), specifically VEGF-A normally promotes vascular proliferation (Coultas et al., 2005), however it is strongly upregulated under oxidative stress (Rossino et al., 2020).

### 3.2 Clinical presentation

AMD can be clinically classified into five stages. Stage 1 manifests as small drusen deposits that are less than 63 μm in diameter and is typically diagnosed at routine optometry visits. In Stage 2, drusen deposits become larger than 63 μm, but smaller than 125 μm. Stage 3 is classified as ‘intermediate AMD’, where drusen deposits become over 125 μm and the retinal pigment epithelium (RPE) begins to show abnormalities. Patients with early to intermediate AMD typically perform well on high contrast, high luminance best-corrected visual acuity tasks (Pondorfer et al., 2019). Interestingly, low luminance tasks show the most pronounced effects of visual impairment in early AMD (Chandramohan et al., 2016; Owsley et al., 2016; Nigalye et al., 2022). This shows the importance of parafoveal rod vision, which can precede inner macular cone loss, resulting in scotopic visual acuity deficits (Nebbioso et al., 2014). Stage 4, or advanced AMD shows RPE damage and geographic atrophy of photoreceptors. The final stage is the transition from non-neovascularized (dry) to neovascularized (wet) AMD (Ferris et al., 2013). Patients with severe AMD can eventually experience bilateral macular vision loss. Complete loss of macular vision affects 15–20 degrees of the visual field (Kolb, n.d.). From the age of 65 to 80 the prevalence of developing intermediate, geographic, and neovascular AMD increase from 5.4 to 23.6%, 0.3 to 6.9%, and 0.4 to 8.2%, respectively (Friedman et al., 2004; Yonekawa and Kim, 2015).

Significant visual impairment occurs with progression to either the geographic atrophy or neovascularized forms of AMD (Figure 1, right middle & bottom). Vision loss following diagnosis of neovascular AMD is rapid (Munk et al., 2012) leading to a rapid decline in quality of life. In one study, patients with vision limited to light perception stated they would be willing to trade 60% of their remaining lifespan to regain perfect vision (Brown et al., 2000). Clearly, the AMD population stands to greatly benefit from vision restoration technologies, yet most vision restoration research has been focused on retinitis pigmentosa (see Table 1).

**Table 1:** Current state of optoelectronic and optogenetic vision restoration.

Name	Group/company	Type	Disease	Outcome
Argus 1	Second Sight Medical Products	Optoelectronic: Epiretinal	RP	- Elicited phosphenes.
IMI	IMI Intelligent Medical Implants	Optoelectronic: Epiretinal	RP	- Elicited phosphenes.
Epi-ret-3	Optoelectronic: Epiretinal	Optoelectronic: Epiretinal	RP	- Only assessed for safety.
Argus II	Second Sight Medical Products	Optoelectronic: Epiretinal	RP	- FDA approval. - 9/13 patients had light perception after 10 years.
IRIS V1	Pixium Vision SA	Optoelectronic: Epiretinal	RP	- Elicited phosphenes.
IRIS V2	Pixium Vision SA	Optoelectronic: Epiretinal	RP	
Alpha AMS	Retina Implant AG	Optoelectronic: Subretinal	RP	- Light perception was observed in 13/15 patients. - Grating acuity: 0.1 to 3.3 cycles per degree.
Alpha IMS	Retina Implant AG	Optoelectronic: Subretinal	RP	- Grating acuity: 0.1 to 3.3 cycles per degree. - 86% were able to perceive light.
Bionic Eye	Center for Eye Research Australia	Optoelectronic: Suprachoroidal	RP	- Above chance performance on Basic Assessment of Light and Motion (BaLM) test - 2.62 logMAR visual acuity
RTx-015	Ray Therapeutics	Optogenetics	RP	- No results posted
RST-001	AbbVie	Optogenetics	RP	- Only assessed for safety.
RESTORE	Nanoscope Therapeutics Inc	Optogenetics	RP	- No results posted
PIONEER	GenSight Biologics	Optogenetics	RP	- No results posted
vMCO-I	Nanoscope Therapeutics Inc.	Optogenetics	RP	- No results posted
PRIMA	Pixium Vision SA	Optoelectronic: Subretinal	AMD	- Prelim results in 5 patients showed improved light perception. - No adverse effects on peripheral vision.

In the absence of vision restoration therapies, a large effort has been focused on slowing the progression of AMD. Given the apparent role of inflammation and immune response in AMD, multiple classes of anti-inflammatory drugs have been tested on AMD, namely, corticosteroids, nonsteroidal anti-inflammatory drugs, immunosuppressants, and biologics (Wang et al., 2011), however, there has been little success in slowing the progression of dry AMD and to date there is no standard treatment. Currently, anti-VEGF medications such as, ranibizumab and bevacizumab are the only available treatments for patients with neovascular AMD, and these have good efficacy at slowing the progression of CNV and prolonging visual acuity (Pieramici and Rabena, 2008). However, in six clinical trials for anti-VEGF medication 30–98% of patients eyes still developed macular atrophy at follow-up (Foss et al., 2022). Because these drugs do not fully prevent the disease from progressing to vision loss, there exists a strong need for vision restoration technologies in AMD, despite the success of disease slowing therapies.

## 4 Vision restoration and disease differences

Optoelectronic prosthetics have been under development since the 1990’s, however, only one device, the now defunct Argus II (Second Sight, Sylmar CA), has received FDA approval under a humanitarian device exemption. These devices were implanted between 2007 and 2019 (Ramirez et al., 2023). While hailed as a breakthrough, the low acuity of restored visual percepts and invasive nature of the device precluded use in the much larger AMD patient population. The fundamental technologies for optoelectronic vision restoration have since evolved to greatly decrease effective pixel size, promising higher acuity of restored vision. The Pixium Prima device (Science Corporation, Alameda CA), offers a theoretical acuity of up to 20/440 (Palanker, 2021), and is currently the only optoelectronic vision restoration technology currently in clinical trials. Moreover, it is the only vision restoration technology of any kind targeting AMD in clinical trials. Despite these engineering advances, several factors limit the wide adoption of retinal prosthesis. First, electrodes still cannot be made small enough to match photoreceptor resolution. Second, optoelectronics can be bulky and require invasive surgery. Third, optoelectronics in AMD patients may create off-target stimulation, since electrical stimulation could activate spared retina surrounding geographic atrophy. Finally, from a business standpoint, retinal prosthetics are complex and invasive implantable medical devices, with a high cost for development and regulatory approval. For a detailed review on the current state of optoelectronics please see (Ayton et al., 2019).

The rapid development of optogenetic technologies over the last decade has fueled innovation in genetic vision restoration strategies. This approach has garnered much excitement due to the simplicity of the design, and the visual acuity it could theoretically achieve, although it does carry the complexity and risks common to any gene therapy, which are not insignificant (Lindner et al., 2022). In addition, given most non-native light sensing mechanisms cannot match the exquisite sensitivity and dynamic range of our native photoreceptors, external hardware in the form of light amplification goggles, are likely to be required to achieve vision across a range of common conditions. This adds additional cost and complexity to optogenetic strategies for vision restoration. There are currently five ongoing clinical trials assessing the feasibility and safety of optogenetics in RP patients, but none in AMD patients. In theory optogenetics could be utilized in both RP and AMD patients, however, there are several factors that may limit feasibility in AMD. Since AMD degeneration is limited primarily to the macula with vision often remaining in the periphery, controlling the spread of gene delivery will be important to prevent off target deleterious effects in spared retina (Simunovic et al., 2019). In addition, viral gene delivery can result in increased inflammatory processes, which are a leading factor already present in AMD; thus, they may exacerbate the effects. Finally, light sensitive proteins need to be continuously produced for the duration of the patient’s life, but the durability of long-term protein expression with gene therapy vectors remains unknown.

**Figure 4:**
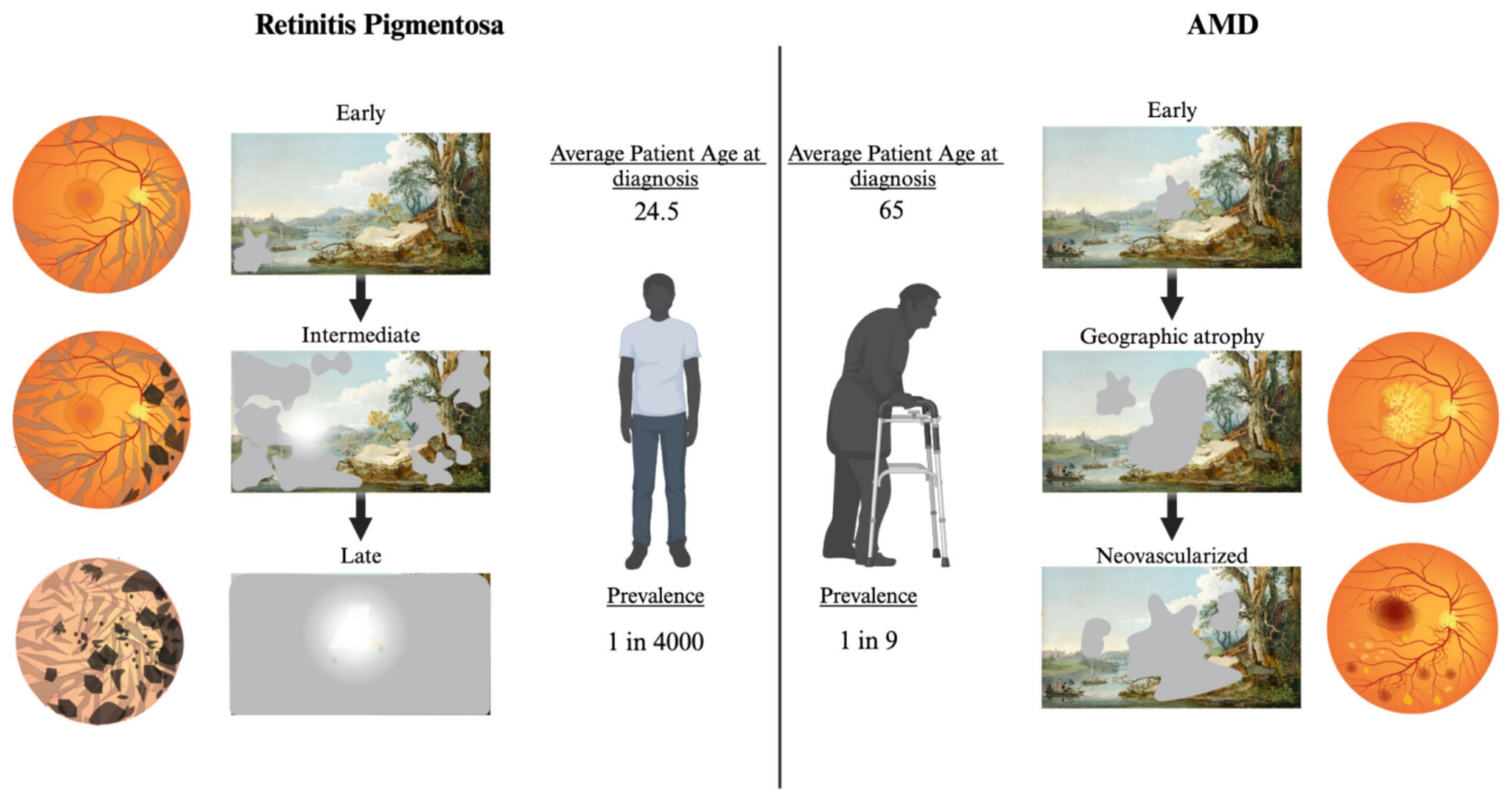
Fundoscopic images throughout disease progression with correlated functional changes for RP and AMD. Left: Progression of RP. In early RP bone spicules form in the outer retina, minimal vision changes. Intermediate RP shows progression of spicules and pigmentary deposits, patients experience night blindness and tunnel vision in the day. Progression to late-stage RP includes dramatic pigmentary deposits, thin vasculature, and optic disk discoloration. Patients are left with minimal photopic vision. Right: Progression of AMD. Early AMD fundus shows development of drusen in the macular region. Scotomas may be present, but patients often do not notice. Progression to geographic atrophy results in larger drusen and noticeable central vision loss. Neovascularized AMD is the most significant loss of central vision. Blood and serum leakage into the subretinal space results in significant photoreceptor death. Created with www.biorender.com.

RP and AMD are significantly different in the way they present and progress clinically. For example, the age of onset for RP is younger than AMD, with patients beginning to show the RP phenotype around 35 years of age (Tsujikawa et al., 2008), however early onset could begin as young as 7.6 years of age (Kaplan et al., 1990). The risks of developing AMD begin increasing at age 65 and the incidence of progression to severe AMD doubles by 75 (Figure 4). Another significant difference between RP and AMD is that rods progressively degenerate from the periphery inward in RP. In early stages, patients experience night blindness advancing to tunnel vision. In final stages, cones degrade secondary to rods leading to the loss of foveal vision resulting in bare or no light perception. In contrast, AMD patients lose rod and cone foveal and parafoveal vision first, and maintain significant peripheral rod dominated vision. The differences in the age of onset of the diseases as well as the quality of vision lost, could ultimately drive differences in the motivation of patients to obtain vision restoration. Differences in the desire to seek vision restoration therapies between RP and AMD patients, particularly when invasive or risky, should also be considered when determining potential target populations.

In both approaches structural remodeling of inner retinal neurons following photoreceptor degeneration may cause changes in network processing. Functional changes in the network, regardless of structural changes, could significantly change the way optogenetic or optoelectronic stimuli are encoded by the network. Structural plasticity has been studied in both RP and AMD retinas prior to and throughout degeneration. However, the effects of structural remodeling have not yet been directly tied to functional changes in circuit processing. Given that detailed functional studies in humans are not feasible, animal models are required to study circuit processing changes following degeneration. RP has many mouse models that recapitulate the disease state and allow for thorough investigation of retinal circuitry, although there are few AMD models and none that fully recapitulate the disease state. As such, much of the assumptions about functional plasticity in AMD has been inferred from RP research.

### 4.1 Structural plasticity

Structural plasticity occurs throughout the nervous system; especially after injury and when presynaptic inputs are lost (Butz et al., 2009). In both RP and AMD structural plasticity begins before complete degeneration of photoreceptors (Jones et al., 1995; Fariss et al., 2000; Phillips et al., 2010; Jones et al., 2003). As the retinal environment becomes unhealthy, reactive gliosis occurs to prevent further retinal damage and promote repair. However, the primary structural glial cells of the retina, Müller glia, also undergo reactive gliosis resulting in hypertrophy and glial scarring (Graca et al., 2018). Glial scarring can be a significant hurdle to subretinal prosthetic vision restoration as it creates an additional barrier between stimulating electrodes and target neurons (Graca et al., 2018). In addition, as photoreceptors degenerate and second order neurons lose their connections, dendrite retraction occurs which is followed by neurite expansion in search of new presynaptic targets. Generally, RP and AMD both exhibit similar structural changes after degeneration of photoreceptors, despite their difference in causes (Jones et al., 1995). This may indicate that loss of input and not disease origin is a major driver of structural plasticity, however this question is far from settled.

Jones et al. (1995) have provided an extensive categorization of the structural changes in RP between human and animal model retinas. Primary hallmarks of structural remodeling after degeneration include second order neuron dendrite retraction, bipolar cell death, second order neurite expansion, and gliosis. While the severity of remodeling depends on each RP variant, the basics of remodeling remain the same. As rods degenerate, rod bipolar cells retract their dendrites and form new contacts with remaining cones, prolonging signaling (Peng et al., 2000). As cones begin to degenerate, all bipolar cells retract their dendrites and send ectopic processes into the inner plexiform layer (Phillips et al., 2010). Cone death initiates Müller glia hypertrophy and the formation of a glial scar. Müller glial cell bodies also migrate into the inner nuclear layer. Both GABAergic and glycinergic amacrine cells have also been found to ectopically migrate out of the inner nuclear layer. Finally, once cells within the inner nuclear layer lose all inputs from photoreceptors, they begin sending neurite projections both into the inner plexiform layer and outer nuclear layer (Jones et al., 2016). These projections seem to have no specificity in their targets and the functional impact of these aberrant connections on signal encoding has not yet been explored.

In addition, the primary glutamate receptor on rod bipolar and ON-cone bipolar cells (mGluR6) also significantly decreases with degeneration in a mouse model of RP (Puthussery et al., 2009). Receptor changes could be deleterious to vision restoration strategies. However, most vision restoration strategies target cells in the inner nuclear or ganglion cell layer and loss of mGlur6 on ON-cone dendrites will have little effect on encoding of prosthetic stimuli.

Comparative studies have shown that RP and AMD human retinas exhibit similar gross structural remodeling after degeneration. AMD retinas exhibit gliosis of Müller cells, translocation of glycinergic amacrine cells, and ectopic processes of GABAergic amacrine cells (Jones et al., 2016; Sullivan et al., 2003). This remodeling is especially severe directly over drusen deposits, but there is extensive remodeling in areas where no drusen deposits are found, suggesting the degree of remodeling is not restricted to the bounds of macular drusen formation. The progression of remodeling is also noticeably faster than in RP and occurs even when rod photoreceptors are still present (Jones et al., 2016). This may indicate separate mechanisms by which structural plasticity is initiated in RP and AMD retinas and indicates that factors beyond loss of inputs contribute to changes. We return to this point of quicker remodeling and its possible impact on functional responses in the sections below.

### 4.2 Functional plasticity

Changes in circuit structure will certainly cause changes in function, but it is also possible to have functional changes in a network beyond what can be seen structurally. Here we define structural plasticity as discrete changes to cell location, dendrite morphology, or receptor number and functional plasticity as changes in the electrical, or biochemical activity within and between cells. There is still a large gap in our understanding of how the retina functionally changes after degeneration (Puthussery and Taylor, 2010). For example, although animal models of RP have been used extensively to characterize physiological responses to prosthetic electrical stimulation or optogenetic stimulation, we still do not fully understand the complexities of how these responses are generated by spared retinal circuitry. In particular, inhibition is a key circuit element shaping retinal response during natural vision, however, few studies have examined if or how inhibitory function is altered following degeneration (Ivanova et al., 2016; Margolis and Detwiler, 2011; Ye and Goo, 2007; Haq et al., 2014), although recent work suggests that it is disrupted (Carleton and Oesch, 2022; Carleton and Oesch, 2024). In this section, we cover the known functional changes of the retina in response to prosthetic stimuli in mouse models of RP and AMD.

Animal models are a powerful tool used to assess circuit function. There are over 10 different mouse models of retinitis pigmentosa; the *rd1* and *rd10* mouse lines being the two most commonly used. Both the *rd1* and *rd10* models of retinitis pigmentosa are valuable models of autosomal recessive retinitis pigmentosa in humans (Chang et al., 2002). Both of these models have mutations in the gene *PDEb6*, which disrupt the function of the beta subunit of phosphodiesterase resulting in excitotoxic cGMP (see Figure 2), similar to common human forms of RP.

Although there are many animal models trying to recapitulate the AMD disease state, due to the diffuse and incompletely understood causes, no one model fully captures all the factors leading to atrophy of photoreceptors (Pennesi et al., 2012). The models that can recapitulate both an ‘AMD like state’ and geographic atrophy are not consistent across all animals, requiring large cohorts and continued genetic modification. Due to these challenges, there has been limited work on the functional changes in the retina in AMD. One recent model that has been used to examine retinal function is laser induced geographic atrophy. While this model does mimic some features of AMD, such as central photoreceptor death and recruitment of the complement pathway, it does not include the chronic inflammatory or pro-angiogenic phenotypes (Khan et al., 2023), which may drive both functional and structural plasticity beyond what is caused by the loss of photoreceptors. This gap in knowledge about retinal function in the AMD disease state presents a significant challenge for the development and implementation of vision restoration strategies in AMD.

#### 4.2.1 Functional changes in RP

One of the best documented functional changes following photoreceptor loss in mouse models of RP is increased spontaneous activity, which tends to be oscillatory in nature. The frequency of the oscillation differs between the two most prominent RP mouse models; 1–2 Hz in the *rd1* model and 4–8 Hz in the *rd10* model (Trenholm and Awatramani, 2015). While the locus of the spontaneous oscillation remains consistent between the two strains, the cause of the different frequencies remains unknown. Prior research has clearly indicated that the gap junction between the AII amacrine cell and ON-Cone Bipolar cell to be necessary for this aberrant spontaneous activity (Ivanova et al., 2016). While the phenomenon has been well documented, the disease mechanisms that cause this change are unknown. One hypothesis is that network hyperpolarization due to deafferentation may play a role, however, the observation that not all RP models show the same phenotype reveals our incomplete understanding (Trenholm and Awatramani, 2015).

Much of the research devoted to understanding aberrant spontaneous activity after degeneration has focused on the deleterious effect on signal to noise for processing restored signals. Spontaneous oscillations will significantly degrade the encoding of prosthetic signals in the retina and in higher visual areas in the brain (Ivanova et al., 2016). Not only do oscillations increase background non-visual noise, but they can also exhibit spatial correlations, potentially disrupting spatial signaling as well (Carleton and Oesch, 2022). This may indicate functional deficits of inhibition that normally limits the spread of information across the retina (Carleton and Oesch, 2022). These functional changes may make restored signals less discriminable, and also confound normal spatiotemporal integration. These challenges would likely exist for both prosthetic and optogenetic restoration strategies.

Additionally, functional changes can be found in the retina’s ability to decompose the visual signal into parallel information channels, such as the ON and OFF pathways that encode the increment and decrement of light, respectively. It was previously shown in normal animals that ON and OFF RGC’s have distinct excitation to inhibition (E/I) ratios in response to light stimuli (Murphy and Rieke, 2008; Pang et al., 2003). We recently investigated the E/I ratios in both *rd10* and *wt* animals in response to electrical stimulation. We found that E/I ratios were broadly similar between *rd10* and wt, however there was significantly more presynaptic inhibition in the OFF pathway of *rd10* retinas compared to wt. How this will impact restored visual signals remains unknown, however, a significant challenge to any vision restoration strategy is the restoration of both ON and OFF signaling pathways, which must work together for normal vision. While it is easy to artificially generate excitation in the presence of light to recapitulate the ON pathway, creating an artificial mechanism to generate excitation in the OFF pathway in response to decrements of light remains a persistent challenge for both prosthetics and optogenetics. Taken together these considerations demonstrate the importance of examining functional changes after degeneration of photoreceptors across disease models.

#### 4.2.2 Functional changes in the geographic atrophy model for AMD

In the laser induced GA mouse model, lesions to the pigment epithelium resulted in GA with similarities to AMD patients. Photoreceptor degeneration presents quickly in this model akin to that observed in patients with CNV. To date, ERG analysis has been used to confirm a reduction in the A-wave (photoreceptors) and increased B-wave (inner retina), but more detailed studies assessing retinal function MEA analysis or single cell electrophysiology following GA have not been done.

Alternatively, there have been studies assessing the impacts of local and acute photoreceptor degeneration on inner retinal circuitry, while not trying to recapitulate the ‘full AMD state’. To induce local degeneration, Denlinger et al. (2020) placed an electrode array in healthy retina, separating the photoreceptor layer from the pigment epithelium. This resulted in photoreceptor death over the implant presumably due to nutrient loss. Retinal responses recorded on an MEA displayed increased spontaneous activity in RGC’s mediated by increased retinoic acid (Denlinger et al., 2020). This hyperexcitability was limited to ganglion cells and did not seem to be originating from the same circuit found in RP mouse models (Trenholm and Awatramani, 2015; Telias et al., 2019). These findings suggest that acute induction of local degeneration may not be equivalent to progressive global degeneration.

At a finer scale, in an acute rod photoreceptor ablation paradigm, the healthy retina was able to functionally compensate for the loss of 50% of rods, inner retinal inhibitory signals were dampened as a compensatory mechanism to maintain rod bipolar voltage output and cone signaling remained unaffected (Care et al., 2020). These results indicate that the retina is capable of fast compensation and plasticity when photoreceptors are lost, however the extent to which acute ablation studies are representative of the full AMD disease state is unclear. As discussed above, structural plasticity begins occurring in disease before all rods are lost in both AMD and RP, and as such acute ablation would not include these changes. Nonetheless, these results aid in our understanding of reactive changes within the retina after photoreceptor loss.

## 5 Discussion

In this review, we compared RP and AMD through the lens of vision restoration. Despite the much greater prevalence of AMD, RP has received far more attention both in preclinical and clinical work regarding vision restoration therapies. While the hope may be that successful vision restoration technologies for RP can eventually be translated to AMD patients following validation of safety and efficacy, there is little functional neural work in AMD to determine if similar outcomes could be expected between RP and AMD. It may be possible that the quick nature of photoreceptor degeneration in AMD will limit the functional changes in the retina, reducing the need to accommodate for circuit dysfunction, such as decreased signal to noise and increased inhibition of the OFF pathway, however, the existence of structural changes prior to photoreceptor degeneration in AMD indicates a more complex scenario. Clearly, more work is needed to explore these ideas.

While patient welfare is the primary goal in the development of vision restoration technologies, commercialization and economic impact are nonetheless important to their success as a business model, which ultimately leads to patient access. The larger population of patients with AMD may result in a more sustainable business model, as many prior prosthetics ventures for RP patients have failed due to the high cost of development and a small patient pool. Furthermore, the Argus II provides the only evidence for an approved implant and was only approved for use in patients with bare or no light perception limiting the number of patients within criteria. While cost-effectiveness increased with implantation, and societal willingness to pay for the Argus II was below the threshold of maximal payment (Vaidya et al., 2014), the economic impact of the Argus II was still low. In the US alone, the economic value of the Argus II, if all RP patients with little to no light perception were implanted, would have been $200 million. However, the true economic value was only $12.8 million, with $100 million of federal funding invested (Walsh et al., 2018). Given the huge cost for bringing either an optogenetic, or optoelectronic device to market, the benefit for implementation of a common strategy across both AMD and RP is clear.

These considerations motivate a need for the development of more animal models in AMD and more comparative preclinical work between RP and AMD. Development and validation of new animal models can be time consuming. Therefore, before new AMD models come online, we may be able to leverage the extensive catalogue of RP models to shed new light onto vision restoration in AMD. For example, given the difference in time course between RP and AMD in humans, we could use existing RP models (Chang et al., 2002) with different degeneration time courses to examine how disease time-course affects function. We may be able to translate these findings to vision restoration strategies in AMD. By cataloging how blind retinas respond to a signal restoration in a broad variety of models of degeneration we can strategically design and implement stimulation parameters based on grouped disease phenotypes rather than a ‘one-size-fits-all’ manner. This should deliver the best quality vision restoration to a broader population in need.
